# Antibodies to post-translationally modified insulin as a novel biomarker for prediction of type 1 diabetes in children

**DOI:** 10.1007/s00125-017-4296-1

**Published:** 2017-05-20

**Authors:** Rocky Strollo, Chiara Vinci, Nicola Napoli, Paolo Pozzilli, Johnny Ludvigsson, Ahuva Nissim

**Affiliations:** 1 Endocrinology & Diabetes, Università Campus Bio-Medico di Roma, Via Alvaro del Portillo 21, 00128 Rome, Italy; 2 Centre for Biochemical Pharmacology, William Harvey Research Institute, Barts and The London School of Medicine and Dentistry, Queen Mary University of London, Charterhouse Square, London, EC1M 6BQ UK; 3 I.R.C.C.S. Istituto Ortopedico Galeazzi, Milan, Italy; 4 Centre for Immunobiology, the Blizard Institute, Barts and The London School of Medicine and Dentistry, Queen Mary University of London, London, UK; 5 Division of Pediatrics, Department of Clinical Experimental Medicine, Medical Faculty, Linköping University, Linköping, Sweden

**Keywords:** Biomarker, Insulin, Insulin autoantibodies, Islet autoantibodies, Oxidative stress, Post-translational modifications, Type 1 diabetes

## Abstract

**Aims/hypothesis:**

We have shown that autoimmunity to insulin in type 1 diabetes may result from neoepitopes induced by oxidative post-translational modifications (oxPTM). Antibodies specific to oxPTM-insulin (oxPTM-INS-Ab) are present in most newly diagnosed individuals with type 1 diabetes and are more common than autoantibodies to native insulin. In this study, we investigated whether oxPTM-INS-Ab are present before clinical onset of type 1 diabetes, and evaluated the ability of oxPTM-INS-Ab to identify children progressing to type 1 diabetes.

**Methods:**

We used serum samples collected longitudinally from the ‘All Babies in Southeast Sweden (ABIS)’ cohort tested for the gold standard islet autoantibodies to insulin (IAA), GAD (GADA), tyrosine phosphatase 2 (IA-2A) and zinc transporter 8 (ZnT8A). We studied 23 children who progressed to type 1 diabetes (progr-T1D) and 63 children who did not progress to type 1 diabetes (NP) after a median follow-up of 10.8 years (interquartile range 7.7–12.8). Of the latter group, 32 were positive for one or more islet autoantibodies (NP-AAB^+^). oxPTM-INS-Ab to insulin modified by ^•^OH or HOCl were measured by our developed ELISA platform.

**Results:**

Antibodies to at least one oxPTM-INS were present in 91.3% of progr-T1D children. oxPTM-INS-Ab co-existed with GADA, IA-2A, IAA or ZnT8A in 65.2%, 56.5%, 38.9% and 33.3% progr-T1D children, respectively. In addition, oxPTM-INS-Ab were present in 17.4%, 26.1%, 38.9% and 41.6% of progr-T1D children who were negative for GADA, IA-2A, IAA and ZnT8A, respectively. ^•^OH-INS-Ab were more common in progr-T1D children than in NP-AAB^+^ children (82.6% vs 19%; *p* < 0.001) and allowed discrimination between progr-T1D and NP-AAB^+^ children with 74% sensitivity and 91% specificity. None of the NP-AAB^−^ children were positive for oxPTM-INS-Ab.

**Conclusions/interpretation:**

oxPTM-INS-Ab are present before the clinical onset of type 1 diabetes and can identify children progressing to type 1 diabetes.

## Introduction

Type 1 diabetes is characterised by insulin deficiency and hyperglycaemia due to extensive destruction of insulin-producing beta cells. The autoimmune nature of the disease is suggested by the presence of a pool of circulating autoantibodies against beta cell proteins even years before the clinical onset, such as autoantibodies to insulin (IAA), GAD (GADA), tyrosine phosphatase (IA-2A) and zinc transporter 8 (ZnT8A) [1].

The mechanism underlying the breach of immune tolerance to beta cell antigens is still unclear. Neoepitopes that are post-translationally modified from the native antigens have been recently described in type 1 diabetes [2–7]. Being different from the native proteins, the post-translational modification (PTM) of self-antigens may be recognised as foreign and result in breakdown of tolerance [8–10]. Oxidative stress is a key feature of many autoimmune diseases and results in an excess of reactive oxidants able to generate oxidative PTM (oxPTM) [4, 11]. Products of oxidative stress are increased in type 1 diabetes [12] and also in individuals at risk [13]. During insulitis, the beta cells that are under stress and the high influx of metabolically active immune cells generate large quantities of reactive oxidants, including the superoxide radical, hydroxyl radical (^•^OH), hypochlorous acid (HOCl) and peroxynitrate [14]. We have shown that autoimmunity to insulin may result from neoepitopes induced by oxPTM [11]. We found that antibodies specific to oxidative post-translationally modified insulin (oxPTM-INS) are present in the majority of newly diagnosed individuals with type 1 diabetes and are significantly more abundant than autoantibodies to native insulin (NT-INS) [11].

Islet autoantibodies (IAA, GADA, IA-2A and ZnT8A) represent the most robust approach to identify individuals at risk and to predict progression to clinical disease in those genetically at risk [15] and in the general population [16]. Children with any two autoantibodies may have a risk of 80% for developing type 1 diabetes during childhood or adolescence [15]. However, multiple antibody testing is required for the best prediction. An additional problem is that radiobinding assays (RBAs), the gold standard for islet autoantibodies, have expensive regulatory requirements. These elements introduce additional complexity that may limit the implementation of autoantibody screening. Finally, a significant number of individuals test negative to these markers [17]. Therefore, the development of alternative technologies remains an unmet need. To address this we have developed an ELISA that detects auto-reactivity to oxPTM-INS. We showed that the assay detecting antibodies to oxPTM-INS (oxPTM-INS-Ab) is highly accurate (84% sensitivity, 99% specificity), may detect over 30% of individuals who are negative to the RBA for IAA and, when combined with IAA, identifies 95% of individuals with newly diagnosed type 1 diabetes [11]. However, the predictive potential of oxPTM-INS-Ab is not known yet.

We hypothesise that oxPTM-INS-Ab are present before the clinical onset of the disease. To test this hypothesis, considering the high prevalence of oxPTM-INS reactivity in newly diagnosed type 1 diabetes, we evaluated the ability of this novel autoantibody specificity to identify children progressing to clinical disease. We used serum samples from the ABIS (All Babies in Southeast Sweden) study, a large prospective study in which unselected children from the general population born during 1997–1999 have been followed prospectively with regular evaluation of islet autoantibodies for the development of type 1 diabetes [18, 19].

## Methods

### Participants and serum samples

Serum samples from the ABIS study were obtained and analysed in a blinded fashion for detection of oxPTM-INS-Ab. The ABIS study is a prospective population-based follow-up study which included 17,055 unselected children born between 1 October 1997 and 1 October 1999 in southeast Sweden [18]. Of the screened children, 116 developed type 1 diabetes during the follow-up. In the present study, we tested 51 samples from the 23 children progressing to type 1 diabetes (progr-T1D), collected longitudinally before diagnosis at three different time points (at the ages of 5, 7 and 11 years). Samples were selected where sufficient serum and autoantibody data were available for this study. Only one time point was available from seven progr-T1D children before the collection at the second time point. As controls, we used samples from 63 children of similar age and sex who did not progress to type 1 diabetes over time, including 64 samples from 32 autoantibody-positive children (autoantibody-positive, non-progressing to type 1 diabetes [NP-AAB^+^]) and 31 samples from autoantibody-negative children (autoantibody-negative, non-progressing to type 1 diabetes [NP-AAB^−^]). NP-AAB^+^ children were defined as positive to at least one islet-antibody marker (IAA, GADA, IA-2A or ZnT8A). Informed consent was obtained from parents prior to the collection of blood. The study was approved by the Research Ethics Committees of the Medical Faculties of Linköping University, Linköping and Lund University, Lund, Sweden and by the Ethics Committee of the Università Campus Bio-Medico di Roma.

### ELISA for detection of oxPTM-INS-Ab

Insulin was chemically modified as previously described to generate oxPTM-INS modified by HOCl, ^•^OH and glycation using ribose [11]. Hen egg lysozyme (HEL; Sigma-Aldrich, Milan, Italy) was similarly modified and used as a control antigen. oxPTM-INS encompasses glycated (GLY-INS), ^•^OH-modified (^•^OH-INS) and HOCl-modified insulin (HOCl-INS). An ELISA was performed using NT-INS, oxPTM-INS, control native HEL or control oxidative post-translationally modified HEL (oxPTM-HEL) as targets. Development and calibration of the ELISA is described in our previous publication [11]. ELISA plates (Nunc, London, UK) were coated with 10 μg/ml of modified or native protein in 0.05 mol/l carbonate/bicarbonate buffer (pH 9.6) at 4°C overnight. Plates were then washed three times with PBS. After blocking for 2 h with 5% BSA in 0.5% Tween PBS, 100 μl of 1:200-diluted serum samples in 5% BSA in 0.5% Tween PBS were added to each well, followed by 2 h incubation at room temperature. Plates were then washed with PBS plus 0.1% Tween, followed by three washes with PBS. Anti-human IgG-horseradish peroxidase-conjugated antibodies (Sigma-Aldrich) were then added at 1:1000 dilution in 5% BSA in 0.5% Tween PBS for another 2 h incubation. The ELISA plates were washed, and 100 μg/ml 3,3′,5,5′-tetramethylbenzidine substrate (Sigma-Aldrich) in 100 mmol/l sodium acetate (pH 6.0), was added. Subsequently, the reaction was stopped with 1 mol/l sulphuric acid. The absorbance was measured at 450 nm using a GENios plate reader and Magellan software (Tecan, Reading, UK). The ELISA absorbance values obtained for HEL and oxPTM-HEL were used as background controls that were subtracted from the absorbance values of NT-INS and oxPTM-INS, respectively. In addition, to account for assay fluctuation, binding to insulin, oxPTM-INS, HEL and oxPTM-HEL was tested for each individual sample on the same plate. Each assay included known positive or negative reference control samples. Longitudinal samples obtained from the same individuals were tested on the same plate. Levels of oxPTM-INS-Ab above the 99th percentile of 88 healthy individuals were defined as ELISA cut-off. Intra-assay CV was <8% (*n* = 10). Inter-assay CVs were <10% and <13% for NT-INS and oxPTM-INS-Ab (*n* = 12), respectively.

### Islet autoantibodies

Islet autoantibodies were measured by RBA. IAA were measured according to the method of Williams et al [20], with some modifications [21]. GADA and IA-2A were measured as previously described by Wahlberg et al [19] and ZnT8A (variants ZnT8RA, ZnT8QA and ZnT8WA) were measured as described by Vaziri-Sani et al [22]. Thresholds have been defined as equivalent to the 98th percentile for GADA, IA-2A and ZnT8A, and the 95th percentile for IAA [23]. In the 2005 Diabetes Autoantibody Standardization Programme (DASP), the RBAs for GADA, IA-2A and IAA achieved 76%, 72% and 28% sensitivity, respectively, with 96%, 100% and 100% specificity. In the 2013 Islet Autoantibody Standardization Programme (IASP), the RBA for ZnT8AR, ZnT8AQ and ZnT8WA achieved 50%, 18% and 36% sensitivity, respectively, with 99%, 100% and 93% specificity.

### HLA genotyping

HLA typing and subtyping was performed as previously described [24]. The HLA genotyping revealed the haplotypes *HLA-DQB1*, *-DQA1* and *-DRB1* and these were categorised as susceptibility-associated (S), neutral (N) and protective (P), according to Hermann et al [24]. Susceptibility-associated haplotypes included *DR4-DQ8* (DRB1*0401/*2*/*4*/*5*-DQB1*0302) and *DR3-DQ2* (DQA1*05-DQB1*02), protective haplotypes included *DR2*-*DQ6* (DQB1*0602), *DR11/12/1303*-*DQ7* (DQA1*05-DQB1*0301), *DR7-DQ3* (DQA1*0201-DQB1*0303), *DR14-DQ5* (DQB1*0503), *DR403*-*DQ8* (DRB1*0403-DQB1*0302) and *DR1301*-*DQ6* (DQB1*0603). Other haplotypes were defined as neutral.

### Statistical analysis

Statistical analyses were performed using Prism Software version 6.01 (GraphPad, San Diego, CA, USA). Differences in antibody levels between groups were tested by the Mann–Whitney test. Longitudinal changes in antibody binding were evaluated by the Wilcoxon paired test. To determine predictive discrimination between progr-T1D and control groups, we used the 99th percentile of the healthy individuals as cut-off point absorbance units to construct a contingency table of positive oxPTM-INS-Ab against clinical diagnosis and tested it by Fisher’s Exact Test.

## Results

### Features of the studied population

The characteristics of the study population are shown in Table 1. At the earliest time point studied, the progr-T1D, NP-AAB^+^ and NP-AAB^−^ groups were comparable in terms of sex, while age was slightly higher in the NP-AAB^+^ group (*p* = 0.04). The majority of progr-T1D children (*n* = 18, 78%) had multiple positive islet autoantibodies; two were single-positive. Two children were negative for IAA, GADA and IA-2A; one child was negative for GADA and IA-2A (IAA was not assessed). Only 25% (*n* = 8) of NP-AAB^+^ children had multiple positive islet autoantibodies. HLA typing was available in 43 children; HLA susceptible phenotypes were more common in progr-T1D than in NP-AAB^+^ and NP-AAB^−^ children (*p* < 0.007), while the prevalence of protective phenotypes was similar between the three groups (*p* > 0.07) (Table 2). In the progr-T1D group, median follow-up from the first sample analysed to diagnosis was 5.1 years (interquartile range 3.2–7.7). After a median follow-up of 10.8 years (interquartile range 7.7–12.8), all children in the control groups, either NP-AAB^+^ or NP-AAB^−^, remained free from diabetes (last assessment performed in December 2016).

**Table 1:** Characteristics of the study population

Characteristics	Progr-T1D (*N* = 23)	Children not progressing to type 1 diabetes	
		NP-AAB^+^ (*N* = 32)	NP-AAB^−^ (*N* = 31)
Age at baseline, years	6.17 ± 1.49	7.61 ± 2.42	7.13 ± 2.07
Sex, male	15 (65)	21 (66)	17 (55)
Multiple autoantibodies (≥2)	18 (78)	8 (25)	NA

Data are presented as means ± SD or *n* (%)

The three groups were comparable in terms of sex, although age was slightly higher in the NP-AAB^+^ group than in the progr-T1D group (*p* = 0.04). Eighteen (78%) of progr-T1D and 8 (25%) of NP-AAB^+^ children had multiple positive islet autoantibodies

**Table 2:** HLA-genotypes prevalence in the study population

HLA susceptibility category	Progr-T1D (*N* = 17)	Children not progressing to type 1 diabetes	
		NP-AAB^+^ (*N* = 16)	NP-AAB^−^ (*N* = 10)
SS	8 (47)	3 (18.75)	0 (0)
SN	6 (35)	3 (18.75)	1 (10)
SP	3 (18)	4 (25)	4 (40)
NN	0 (0)	2 (12.5)	2 (20)
NP	0 (0)	2 (12.5)	2 (20)
PP	0 (0)	2 (12.5)	1 (10)

Data are presented as *n* (%)

HLA haplotypes were available in 43 children and were categorized into susceptibility-associated (S), neutral (N) and protective (P) groups according to Hermann et al. [24]. Susceptibility-associated haplotypes included *DR4-DQ8* (DRB1*0401/*2*/*4*/*5*-DQB1*0302) and *DR3-DQ2* (DQA1*05-DQB1*02), protective haplotypes included *DR2*-*DQ6* (DQB1*0602), *DR11/12/1303*-*DQ7* (DQA1*05-DQB1*0301), *DR7-DQ3* (DQA1*0201-DQB1*0303), *DR14-DQ5* (DQB1*0503), *DR403*-*DQ8* (DRB1*0403-DQB1*0302) and *DR1301*-*DQ6* (DQB1*0603). Other haplotypes were defined as neutral

### Cross-sectional evaluation of oxPTM-INS-Ab prevalence

**Fig. 1:**
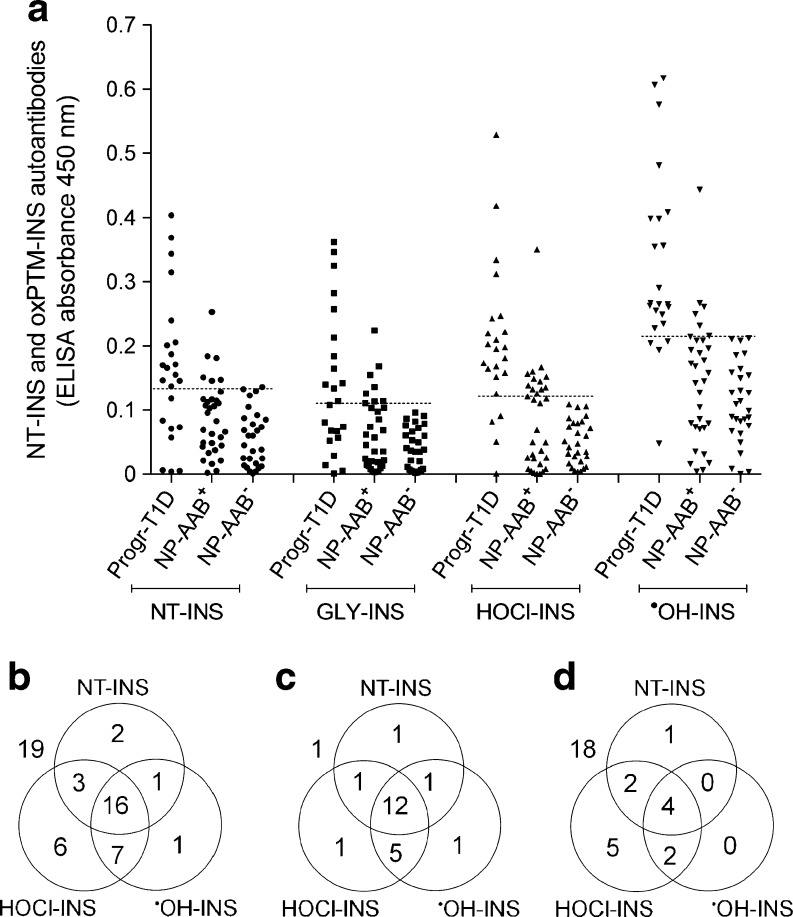
Cross-sectional evaluation of antibody binding to oxPTM-INS in study population. (**a**) Reactivity to NT-INS and oxPTM-INS was significantly higher in samples from progr-T1D children compared with non-progressing children, regardless of whether they were NP-AAB^+^ or NP-AAB^−^ to the standard islet autoantibody markers (*p* < 0.001). Binding to oxPTM-INS modified by HOCl and ^•^OH was significantly higher than to NT-INS in progr-T1D children (*p* < 0.0001). Data on the earliest time point available are reported. Values above the dashed lines were defined as positive for antibodies to NT-INS and oxPTM-INS modified by glycation (GLY), HOCl or ^•^OH, respectively (99th percentile of a group of 88 healthy control children). (**b**–**d**) Overlapping prevalence of antibodies to NT-INS and oxPTM-INS in all children positive for at least one islet autoantibody (**b**) and in progr-T1D (**c**) and NP-AAB^+^ children (**d**). Values outside the circles are children negative for the antibody evaluated in the diagram

Binding and prevalence of oxPTM-INS-Ab were compared between progr-T1D, NP-AAB^+^ and NP-AAB^−^ children at the earliest time point available. In the progr-T1D group, binding to HOCl-INS and ^•^OH-INS was significantly higher than binding to NT-INS (*p* < 0.001; Fig. 1a). Serum samples from 21 (91.3%) progr-T1D children bound to at least one oxPTM-INS (^•^OH-INS or HOCl-INS) and 17 (73.9%) children were found to be double-positive to both ^•^OH-INS and HOCl-INS. Binding to HOCl-INS and ^•^OH-INS was higher in sera from progr-T1D children compared with either NP-AAB^+^ or NP-AAB^−^ children (*p* < 0.001; Fig. 1a). oxPTM-INS-Ab were more common in progr-T1D children than in NP-AAB^+^ children, with respective reactivity to ^•^OH-INS in 82.6% (19/23) vs 19% (6/32) (*p* < 0.0001) and reactivity to HOCl-INS in 82.6% (19/23) vs 40.6% (13/32) (*p* = 0.0024) (Fig. 1b–d). ^•^OH-INS-Ab allowed discrimination between progr-T1D and NP-AAB^+^ children with 74% sensitivity and 91% specificity. The overlap between NT-INS and oxPTM-INS-Ab is shown in Fig. 1b–d. Binding to NT-INS was more common in progr-T1D children than in NT-AAB^+^ and NT-AAB^−^ children (*p* < 0.001). Binding to both NT-INS and oxPTM-INS occurred in 14 (60%) progr-T1D children, while 7 (30%) displayed binding only to oxPTM-INS. In the NP-AAB^+^ children, 6 (19%) displayed binding to both NT-INS and oxPTM-INS, while 7 (22%) displayed binding only to oxPTM-INS. None of the NP-AAB^−^ children were positive for oxPTM-INS-Ab.

### Longitudinal changes of oxPTM-INS-Ab in prog-T1D children

**Fig. 2:**
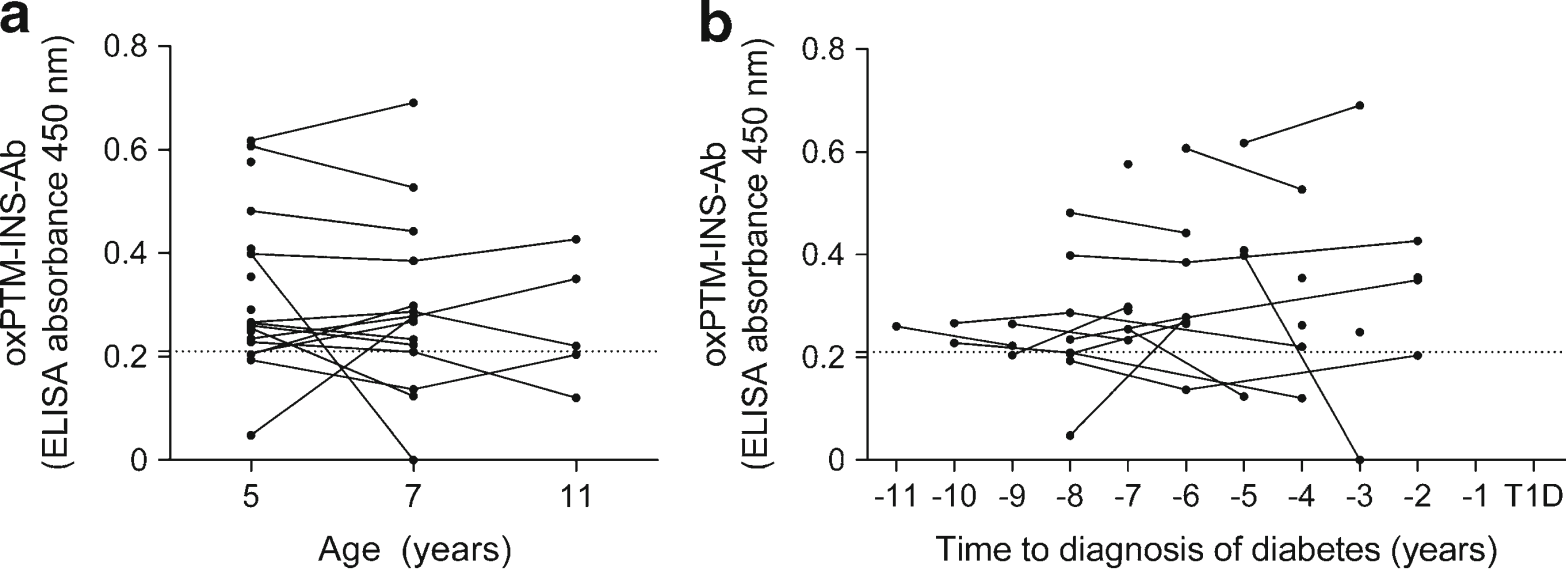
Longitudinal changes of oxPTM-INS-Ab in prog-T1D children according to age (**a**) and time before diagnosis of type 1 diabetes (**b**). Values above the dashed lines were defined as positive for antibodies to oxPTM-INS; ^•^OH-INS is shown as an example for oxPTM-INS-Ab

Binding to oxPTM-INS (either ^•^OH-INS or HOCl-INS) did not change significantly over time (median follow-up 3 years [range 3–6 years]; *p* = 0.725; Fig. 2a). Seroconversion occurred in four children: two became positive at a later stage while two became negative. When evaluated according to time before diabetes onset, reactivity to oxPTM-INS appeared as early as 11 years before disease onset (median time to diabetes onset 6 years [range 2–11 years]; Fig. 2b).

### Distribution of oxPTM-INS-Ab and islet autoantibodies

**Fig. 3:**
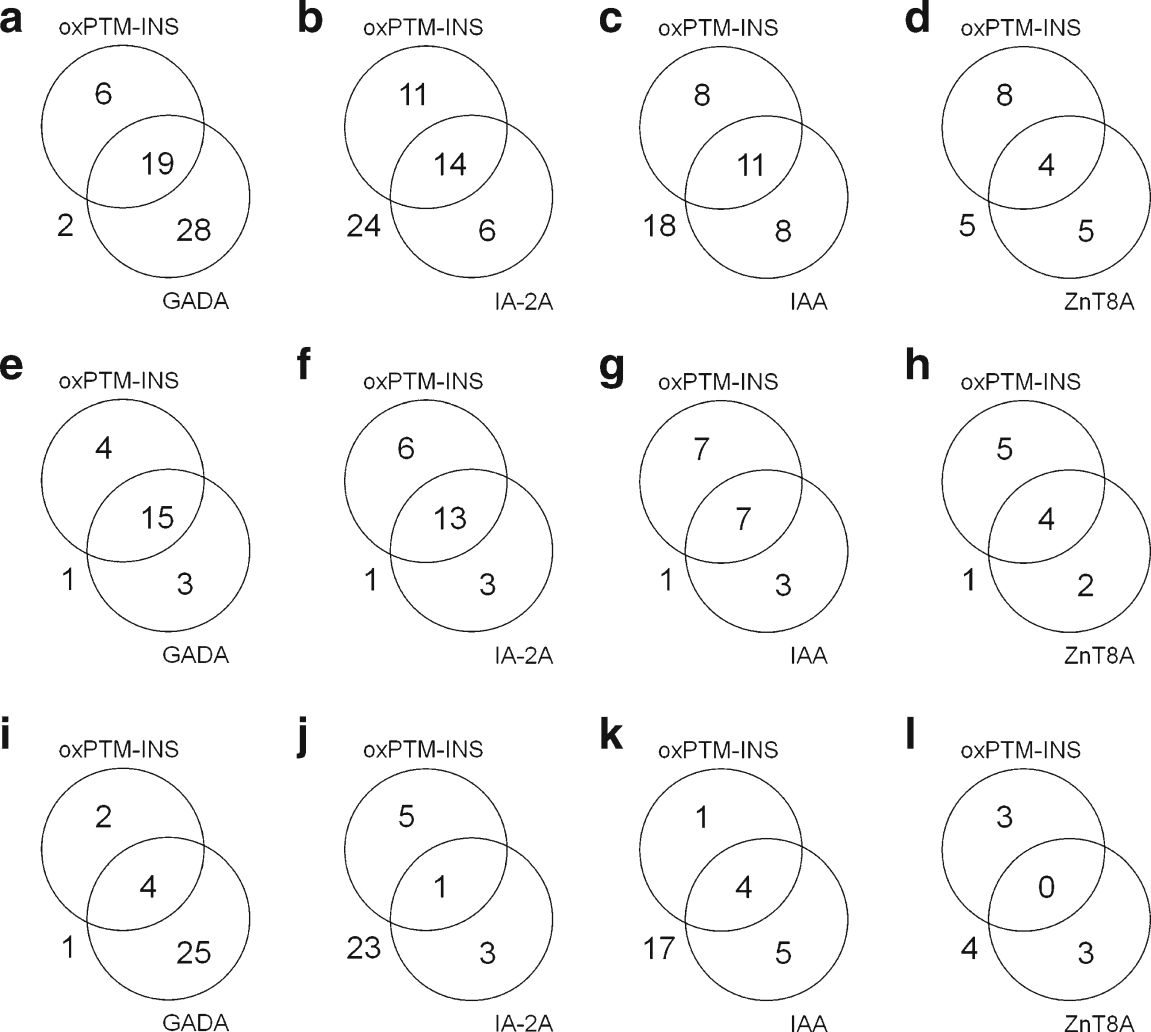
Overlapping prevalence of oxPTM-INS-Ab, GADA, IA-2A, IAA and ZnT8A. Data are shown for the whole study population of children positive to at least one islet autoantibody (**a**–**d**) and for progr-T1D (**e**–**h**) and NP-AAB^+^ children (**i**–**l**). Data for IAA and ZnT8A were available in 45 and 22 children, respectively. ZnT8A included positivity to one or more ZnT8RA, ZnT8AWA and ZnT8QA variants. ^•^OH-INS is shown as example for oxPTM-INS-Ab. Values outside the circles are children negative to the antibody evaluated in the diagram

**Fig. 4:**
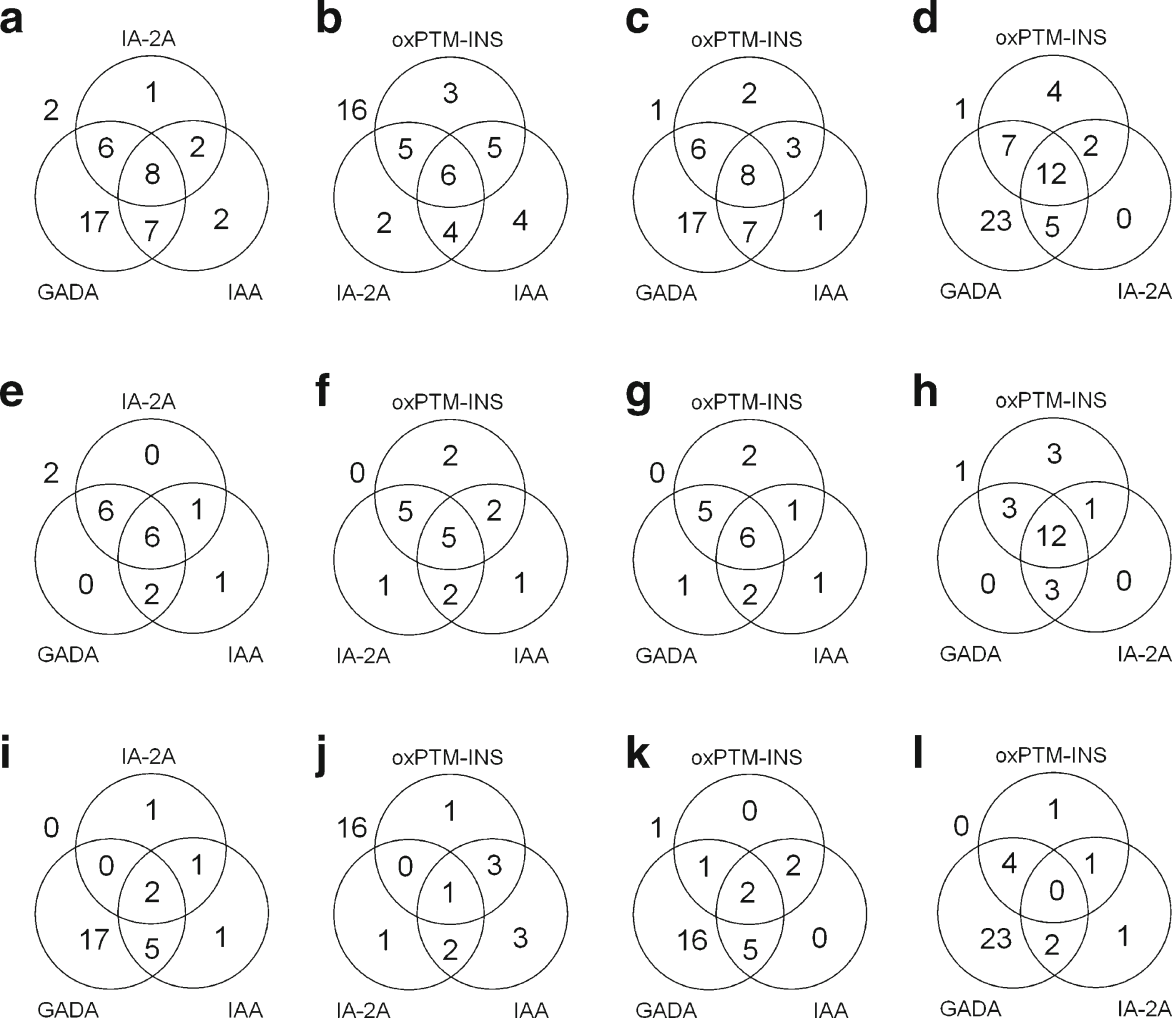
Overlapping prevalence of oxPTM-INS-Ab, GADA, IA-2A and IAA evaluated with three standard islet autoantibodies or with oxPTM-INS-Ab substituted for GADA, IA-2A or IAA. Data are shown for the whole study population of children positive to at least one islet autoantibody (**a**–**d**) and for progr-T1D (**e**–**h**) and NP-AAB^+^ children (**i**–**l**). IAA was not available in five progr-T1D and five NP-AAB^+^ children; therefore these children are not included in the diagrams assessing the overlapping prevalence of IAA. ^•^OH-INS is shown as example for oxPTM-INS-Ab. Values outside the circles are children negative to the antibodies evaluated in the diagram

Figure 3 shows the degree of overlap between oxPTM-INS-Ab (^•^OH-INS-Ab) and other islet autoantibodies in progr-T1D and NP-AAB^+^ children. IAA data were available in 18 progr-T1D and 27 NP-AAB^+^ children and ZnT8A data were available in 12 progr-T1D and 10 NP-AAB^+^ children. While binding and prevalence of oxPTM-INS-Ab were significantly higher in progr-T1D children than in NP-AAB^+^ children (*p* < 0.001), both titres and prevalence of GADA, IAA and ZnT8A were similar between the two groups (*p* > 0.257). IA-2A was the most specific marker among autoantibodies evaluated, being significantly higher in the progr-T1D group than in the NP-AAB^+^ group (*p* < 0.001). In the progr-T1D group, 19 (82.6%) children were positive to ^•^OH-INS-Ab, 18 (78.3%) to GADA, 16 (69.5%) to IA-2A, 10 (55.5%) to IAA and 6 (50%) to ZnT8A (Fig. 3e–h). oxPTM-INS-Ab co-existed with GADA, IA-2A, IAA or ZnT8A in 15/23 (65.2%), 13/23 (56.5%), 7/18 (38.9%) and 4/12 (33.3%) progr-T1D children (Fig. 3e–h). In addition, oxPTM-INS-Ab detected 4/23 (17.4%), 6/23 (26.1%), 7/18 (38.9%) and 5/12 (41.6%) progr-T1D children who were negative for GADA, IA-2A, IAA and ZnT8A, respectively (Fig. 3e–h). Two progr-T1D children were negative for GADA, IA-2A and IAA (ZnT8A not assessed) but showed positive for oxPTM-INS-Ab. The assessment of oxPTM-INS-Ab in combination with IA-2A and IAA led to the identification of 100% progr-T1D children with the lowest percentage of false-positive results among the other possible antibody combinations (Fig. 4). In the NP-AAB^+^ group, oxPTM-INS-Ab reactivity was similar in those who were positive to one or more standard islet autoantibodies (*p* > 0.148).

## Discussion

We have recently shown that oxPTM-INS-Ab are very common in newly diagnosed type 1 diabetes, being detected in 84% of individuals [11]. In this study, we found that oxPTM-INS auto-reactivity is present before diabetes diagnosis in over 90% of individuals. oxPTM-INS-Ab could discriminate between prog-T1D children and those who did not progress to type 1 diabetes regardless of positivity to other islet autoantibodies. Therefore, these data may suggest a potential role for oxPTM-INS-Ab as a predictive biomarker of type 1 diabetes.

The main strength of our study is the prospective evaluation of oxPTM-INS-Ab in a well-characterised cohort of children from the general population, tested for the standard diabetes autoantibodies. The uniqueness of the ABIS study resides in the possibility of applying its findings to the general population, since participants were not selected according to predetermined diabetes risk (genetic or familial). An additional strength was that the long follow-up in some progr-T1D children allowed us to discover whether oxPTM-INS-Ab develop very early in the natural history of the disease. Our study has limitations. The earliest time point tested in the progr-T1D children was at 5 years of age. Data from birth cohorts suggest that peak age of seroconversion in high-risk individuals is around 2 years of age [25]. Therefore, we cannot exclude the possibility that children defined as negative for the standard islet autoantibodies have not previously been seropositive for one or more islet autoantibodies. A second limitation is that the determination of ZnT8A was performed on a limited number of children, as ZnT8A assays became available only after the follow-up for many children in the ABIS study.

Our findings shed further light on type 1 diabetes pathogenesis. Detection of oxPTM-INS reactivity before clinical onset of type 1 diabetes is consistent with evidence that the unbalanced redox state takes place early in the natural history of the disease. Individuals with a short disease duration have an early impairment in antioxidant capacity and over threefold increased levels of lipid peroxidation regardless of blood glucose control [12]. An impaired oxidation status anticipates dysglycaemia; it has been shown that plasma malondialdehyde and erythrocyte malondialdehyde, two markers of oxidative stress, are abnormal in euglycaemic individuals at increased risk for type 1 diabetes compared with individuals not at risk [13]. These data, together with our findings, may support the involvement of oxidative stress in type 1 diabetes pathogenesis.

To our knowledge, this is the first study investigating antibody reactivity to oxPTM/PTM of a beta cell antigen as a predictive biomarker of type 1 diabetes. Therefore, a direct comparison with prediction potential of other modified antigens is not possible. Our data are consistent with previous finding by our group in newly diagnosed individuals with type 1 diabetes. Similar to our previous study [11], oxPTM-INS-Ab identified over 80% of individuals with the disease and were able to detect more than one-third of those who tested negative to the IAA assay. Additional neoepitopes derived from PTM of beta cell autoantigens have been described in humans and in animal models of type 1 diabetes, including antibody response to oxidised GAD [26] and T cell reactivity to GAD citrullination [7], C-peptide deamination [5] and hybrid fused peptides [2, 27]. The response to modified antigens may also involve proteins that are not proper to beta cells [4, 28], especially in the presence of defined genetic background. An example is the identification of antibodies to oxPTM-collagen type II in a large proportion of individuals carrying HLA-DRB1*04 [4]. In this regard, the extracellular matrix surrounding beta cells [29], or other tissues attacked by autoimmune response in type 1 diabetes (thyroid, gut, joints, etc) [30], may become additional potential targets of oxPTM [4, 31]. Often such PTM forms induce a more pronounced immune reactivity than the native antigen.

Our results may also have implications for disease prediction and staging. Consistent with previous findings [15, 32], we found low predictive accuracy of GADA and IAA when analysed as a single test, while IA-2A showed a highly specific association with type 1 diabetes progression [15]. A main finding of our study is the predictive accuracy of oxPTM-INS-Ab. Of note, oxPTM-INS-Ab identified individuals with preclinical disease otherwise classified as antibody-negative or single-positive. As highlighted by a recent statement by the JDRF, Endocrine Society and ADA, islet autoimmunity (as defined by the presence of two or more islet autoantibodies) represents the earliest stage of type 1 diabetes and identifies a target population for prevention trials and future preventive strategies [33]. If confirmed in larger studies, oxPTM-INS-Ab may be adopted as an additional biomarker to further redefine disease taxonomy, allowing better prediction and therefore better stratification into eligibility trials.

In conclusion, we showed that immune reactivity to oxPTM-INS is present before clinical onset of type 1 diabetes and that measurement of oxPTM-INS-Ab may identify children likely to progress to type 1 diabetes. This is the first evidence suggesting that oxPTM of a beta cell autoantigen precedes diabetes onset in humans and that auto-reactivity to oxPTM may act as a predictive biomarker of the disease. Additional studies with larger cohorts are required to confirm the predictive potential of oxPTM-INS-Ab in type 1 diabetes.

## Abbreviations

ABIS: All Babies in Southeast Sweden
GADA: GAD autoantibodies
GLY-INS: Glycated insulin
HEL: Hen egg lysozyme
HOCl-INS: HOCl-modified insulin
IA-2A: Tyrosine phosphatase autoantibodies
IAA: Insulin autoantibodies
^•^OH-INS: ^•^OH-modified insulin
NT-INS: Native insulin
oxPTM: Oxidative PTM
oxPTM-HEL: Oxidative post-translationally modified HEL
oxPTM-INS: Oxidative post-translationally modified insulin
oxPTM-INS-Ab: Antibodies to oxPTM-INS
progr-T1D: Progressing to type 1 diabetes;
NP-AAB^+^: Autoantibody-positive, non-progressing to type 1 diabetes
NP-AAB^−^: Autoantibody-negative, non-progressing to type 1 diabetes
PTM: Post-translational modification
RBA: Radiobinding assay
ZnT8A: Zinc transporter 8 autoantibodies
